# Hospital and Institutional News

**Published:** 1917-10-06

**Authors:** 


### HOSPITAL AND INSTITUTIONAL NEWS.

### THE SHORTAGE IN THE MEDICAL PROFESSION.

In spite of the war the medical schools commenced their winter session on October 1. At Charing Cross Hospital Dr. Addison, the Minister of Reconstruction, who was at one time on the staff of the hospital, distributed the prizes and gave the inaugural address. The main point on which he laid stress was the inevitable shortage in the medical profession. He stated that the annual loss by death and retirement was between 900 and 1,000, and that in 1919 the addition to the numbers of the profession would be only a little more than 500, so that for some years to come the yearly additions would not make up for the yearly losses. Even at the present time there were not sufficient medical men adequately to examine and treat the million children attending the elementary schools of the country. This and other difficulties would have to be met, and if the older methods proved insufficient, new methods would have to be employed. The deficiency in medical men will to a certain extent be relieved by the large number of women who are now studying medicine, but it is obvious that these cannot replace wholly the loss. The number of entries at some of the schools is much less than in peace-time, and many of the examining bodies have for the present relaxed the rule as to the attendance at certain lectures, for in many cases the lecturers are serving with the Forces or are engaged otherwise in treating wounded soldiers.

### THE NEW IMPERIAL ORDER.

Special interest was shown by hospital workers of all ranks in the first investiture in connection with the new Order of the British Empire, for among those who were to be decorated by the King were representatives of their own profession, both on the medical, administrative, and nursing sides. The Hon. Arthur Stanley, of the British Red Cross Society, was invested with the insignia of a Knight Grand Cross of the Order and received the honour of knighthood. In consequence of his lameness, Mr. Stanley was unable to kneel to receive the accolade of knighthood, and the King, with his accustomed consideration, therefore conferred the honour on him separately. Sir Arthur Pearson, the blind chief of St. Dunstan's Hostel, was led in by one of His Majesty's gentlemen ushers to receive his insignia as a Knight Grand Cross. Among the Dames Grand Cross invested were Lady Paget, for her work with the Serbian Relief Fund, and Mrs. Katherine Furse, Commandant-in-Chief of the Women's Voluntary Aid Detachment, while Dr. Flora Murray and Dr. Louisa Garrett Anderson were also recipients of decorations as Commanders. Mrs. Cecil Baker, honorary secretary of the British Ambulance Committee, and Miss E. P. Hughes, of Barry, well known for her work in connection with the Glamorgan branch of the Red Cross Society, were admitted into the Order. Several detachments of nurses waited outside Buckingham Palace to congratulate colleagues and friends. One such band from Endell Street Hospital escorted their decorated doctors home, accompanied by the hospital's mascot dog. The sergeants from the Balgowan Hospital, Beckenham, made a special trip to the Palace in order to congratulate their Commandant, Mrs. J. Fisher. In their hospital blues they lined up as she left the Palace, doffing their hats and cheering lustily. When they surrounded her she passed round for their inspection the gorgeous leather case, embossed in gilt, with the arms and monogram of the Order, containing the decoration.

### THE AURAL BOARD.

The Pensions Minister has set up a special Aural Board for the purpose of dealing with the cases of discharged soldiers who are deaf. The chairman of the Board is Major Dundas Grant, who is consulting surgeon to the Central London Nose, Throat, and Ear Hospital. Some classes for the instruction in lip reading of deafened soldiers have been arranged under the supervision of the Board. The chief class consists of morning and afternoon instruction, the course lasting about fourteen weeks; but in addition to these there are evening classes intended mainly for those whose deafness is less complete, and this class will meet three or four times a week, and it will have the advantage that those who attend it will not have to give up their daily employment. All these classes are to be held at the College of the National Association for the Aural Instruction of the Deaf, at 11 Fitzroy Square, of which Mr. Sibley Haycock is the principal. For the treatment of those cases of deafness in which there is no need to teach lip-reading it has been arranged that special aural clinics shall be held in the evening at hospitals selected on account of their accessibility. At these clinics the ear disease will be treated, and in many cases it is probable that some return of hearing may be obtained.

### THE WORKING OF THE VENEREAL DISEASES ACT, 1917.

Certain important sections of the Venereal Diseases Act, 1917, and Orders issued by the Local Government Board in connection with it, come into force on November 1, and a circular has been issued by the Board to the councils of counties and county boroughs in England and Wales calling attention to the Act and the methods of putting it into operation. Section 1, which deals with gratuitous treatment, depends largely for its successful working upon the activity of local bodies, and an Order of the Board, bringing the section into operation within any area, cannot be made until a scheme for the gratuitous treatment of persons within that area has been approved by the Board and is already in operation. This Order has been made in respect of fifteen counties and twenty-one county boroughs, in which suitable arrangements have been made and approved.

Seemingly unqualified practice, which in the opinion of the Royal Commission "constitutes one of the principal hindrances to the eradication of those diseases," will continue until the other local authorities have completed their arrangements. Happily, Section 2 of the Act, which deals with undesirable advertisements, public notices, and announcements with respect to the treatment of venereal disease, or the prescription of remedies, or the giving of advice in connection with the treatment, does not depend upon the activity of the local councils and comes into full force upon the date named.

### AVERAGE COST PER BED UNDER THE RED CROSS.

A report of the accounts of 982 Red Cross hospitals in England, Ireland, and Wales attempts, for the first time, we believe, to calculate the cost per occupied bed. This return deals with 275,932 patients who, during 1916, were passed through 44,147 beds. The total average daily cost of maintenance per patient is stated to be a fraction of a penny over 3s. 6d. The cost of the controllable expenditure of the general hospitals in London, as shown by the return for the same year of King Edward's Hospital Fund, ranged from 3s. 10d. to 7s. a day per patient. It is very difficult to draw any conclusions from a comparison of the average cost of the two groups of hospitals, chiefly because there are insufficient data in respect of the Red Cross figures. Rent and rates are brought into neither calculation, and the same remark applies to the value of voluntary service, which is quite considerable in connection with Red Cross work. The voluntary hospitals in London value the whole of the gifts received in kind, and bring the figure into the income and expenditure account. Is this the case with Red Cross accounts? The point is important because the war hospitals must benefit very largely by gifts of linen, dressings, furniture, and food. On the other hand, the voluntary hospitals have the benefit of the service of trained officials, who have been perfecting methods of economy for many years. The report referred to states that the cost of maintenance and administration of 982 hospitals was £2,205,085, and that War Office grants amounted to £1,530,144, leaving a voluntary contribution during 1916 of £674,940.

### DR. MARCUS PATERSON.

Dr. Marcus Paterson, who has been in poor health for some time, has been compelled for this reason to resign his position as chief medical director of the Welsh National Memorial Association, which he has held since the initiation of the Association in 1911. No steps have as yet been taken to fill the vacancy, but the General Committee will deal with the situation at its next meeting. Dr. Paterson, whose medical experience has been gained in all parts of the world, is at present suffering from Malta fever. Before accepting any other post it is his intention to take a prolonged rest. Apart from the noteworthy service he has rendered in the campaign against consumption in Wales, Dr. Paterson is known for the excellence of his work as organiser of the Frimley Sanatorium. He has made tuberculous disease his special study, and published several books on various aspects of it.

### POOR-LAW INFIRMARIES AND SOLDIERS' WIVES.

Mr. J. L. Goldspink, clerk to the Lambeth Board of Guardians, has interviewed Mr. J. S. Oxley, inspector of the Local Government Board, with reference to the notification to the Army Council of the names of soldiers' dependents in receipt of allowances who are admitted to Poor-Law infirmaries, referred to in the last issue of The Hospital, page 521. He informed the Guardians that there was nothing said in the interview which would deter the Board from declining to give the information to the Army Council, and acting on this the Guardians have now decided to refuse to notify them of these cases. Mr. Frank Briant, L.C.C., the chairman, said in one instance which came under his notice the allowance to a soldier's wife was stopped for six weeks, although the child was only ten days in the infirmary. The Army Council have stated that when these allowances are stopped the recipients have the option of applying to the Local Pensions Committee for sufficient money to tide them over whilst the dependent is in the infirmary. Unfortunately, there must be considerable delay ere the committee's investigations into each case are made, with the result that the recipients of the allowances are almost bound to suffer some hardships. An effort is to be made through the intervention of the Metropolitan members of the House of Commons to get the Army Council to withdraw their rule in these particular cases, and it is anticipated that they will be supported by the great majority of the Boards of Guardians, not only in London, but throughout the country.

### AN UNCOMFORTABLE HOSPITAL.

If we are to accept literally the description of life in an American war hospital in connection with a training camp, as it is described in an article on military medicine in the *Medical Record* of New York, it appears that in several particulars the comfort of the patient is subservient to military ideas of discipline. We are told that at seven o'clock every patient who can has to get out of his bed and must leave the ward until inspection, which lasts until nine o'clock, is over. The toilet accommodations are closed during inspection-time, which, as the writer says, is rather rough in some cases. Another little diversification of the hospital sergeant or orderly is the exact alignment of cots; this is done by a series of kicks, jerks, and pushes till from one end of the ward to the other each cot stands on the exact line. It is an operation in which two orderlies take part, one sighting down the line and the other doing the "rough stuff," and the effect on a man who happens to be suffering from a raging headache can be easily imagined. When a doctor is commencing his military duties he is generally advised "to learn to pass the buck." This is by no means a joke, it is a real underlying factor in an officer's advancement. The expression means that he must never be caught being responsible for anything. For at the monthly inspection all deficiencies in the matter of instruments, etc., are noted by the superior inspector, and the doctor in charge of the case has to make good the loss out of his own pocket, and that with little delay, because the Government takes care to deduct such charges from his salary.

### FILMING A COLLIERY "ACCIDENT."

The committee of the Chesterfield and North Derbyshire Hospital may well congratulate themselves on the result of the four days' bazaar organised for the purpose of extinguishing the debt of £4,090 on the hospital. A series of efforts under the auspices of the Chesterfield Chamber of Commerce, including a Civic and Industrial Exhibition, had already resulted in £1,000 being raised, and the bazaar, including donations, realised close on £7,000. A novel feature in connection with the enterprise was the filming of an "accident" at one of the local collieries, further pictures showing the ambulance on the road, its arrival at the hospital, scenes in the casualty department, wards, and theatre, and finally the departure of the "accident" convalescent. The film was exhibited at all the cinema houses in the district, when collections were taken and the date of the bazaar placed on the screen. A fancy-dress dance for children and a ball will bring the effort to a close, and it is hoped that the total amount to be handed over to the hospital will reach £10,000.

### INCREASED ACCOMMODATION FOR PATHOLOGICAL WORK.

Owing to the great increase of the pathological work at Lambeth Infirmary, Dr. Lionel Baly, the medical superintendent, asked the Board to make provision for additional accommodation. This they agreed to do, at his suggestion, as he pointed out that the work could not now be done in the one room provided. Under present conditions the outdoor work was dealt with by one assistant medical officer, and therefore a consulting-room could not be utilised for pathology. The Guardians have adopted Dr. Baly's proposal, so that it is anticipated that the increase in the pathological work will be adequately dealt with in the future. Pathology forms such an important part of the work of infirmaries and hospitals at the present time that due facilities must be given to medical officers for carrying out this work, if their results are to be of the highest value.

### A MEDICAL EYE-WITNESS ON THE EAST AFRICAN CAMPAIGN.

When a writer of a book on a little known aspect of the war is a medical man who was before the war a practised writer the hospital world will be anxious to know what he has to say. Such is the case with Captain F. Brett-Young, who has published a work on the East African campaign, in which he took part as a medical man attached to the 2nd Rhodesian Regiment, before joining the Indian ambulance unit. Having served under General Smuts when that veteran was driving the Germans down the Tanga railway in 1916, he calls his book "Marching on Tanga." An attack of fever gave him leisure to write it in hospital, but, like another Carlyle, he had his manuscript destroyed (by a torpedo), and in the end, like the author of the "French Revolution," he had entirely to rewrite his book. Kilimanjaro, the Fuji of South Africa, looms through his pages, as it looms through the spaces of the country, and lends a grandeur to the scene in which the story unfolds. No book was more needed than one on the East African campaign. We are fortunate that Messrs. Collins have published a volume which the trained mind of a medical man and the practised pen of a man of letters have combined to make worthy of its subject.

### MEDICAL DRAUGHTSMEN.

John Leech, the centenary of whose birth was recorded a few weeks ago, was, we are told by the writer of an interesting article in the Literary Supplement of the *Times*, intended for a surgeon. He was a Charterhouse boy in the time when the school was within sound of Bow Bells, and later went to learn surgery at St. Bartholomew's Hospital. Here he was associated with another student, who also was to become prominent in the realm of humorous literature—namely, Albert Smith. The family purse not proving long enough, Leech was removed from the medical school and placed with "the ingenious Mr. Whittle of Hoxton," who, under the guise of healer, devoted most of his attentions to pigeons and boxing. This method of learning surgery not proving satisfactory, Leech was next placed with Dr. John Cockle, the son of the pill man, but that arrangement did not last long, and Leech appears to have passed directly to a different form of art, in which he was to discover financial success. It is possible, however, to be a medical man and also to excel with the pencil. The great Charcot added draughtsmanship of a very high order to his other accomplishments, and, to quote another of several instances, Lister was responsible for many of the best illustrations in his own works. Sir Seymour Haden is much better known to us as an etcher than as a physician, and the present money value of one of the products of his needle point is probably greater than several months' fees earned by him as a doctor.

### THE CAVELL PAPERS.

Many people admire Nurse Cavell, but very few know precisely what she did, where, if at all, she was open to criticism, what pretexts the Germans had for her conviction, whether or not a similar sentence had previously befallen a similar act, and, in short, what are the facts on which the verdict of posterity will determine Edith Cavell's place in its esteem. We therefore think it timely to call attention to the article by Mr. Hugh Gibson, the First Secretary of the American Legation in Brussels, which appears in the issue of *Land and Water* published on September 27. It is entitled "The last hours of Edith Cavell." Its writer quotes from a journal composed at the time, and was himself then a neutral whose opinions and acts were, from the nature of his office, as responsible and impartial as human opinions and actions ever are. The gist of what he says is this, which we propose to summarise without distracting comment.

### THE ARGUMENTS OF THE PROSECUTION.

On August 5, 1915, he writes, Miss Edith Cavell, an Englishwoman, directress of a large nursing home in Brussels, was quietly arrested by the Germans on the charge that she had helped stragglers from the Allied armies to cross the frontier from Belgium to Holland, and to this end had supplied them with information, directions, money, and clothes. In answer to inquiries from the American Legation Baron von der Lancken, chief of the Political Department, stated that Miss Cavell was in prison and had admitted that these charges brought against her were true. Since "as a matter of principle" accused persons were not allowed by the Germans to have any interviews whatever, the task of defending Miss Cavell was hopeless from the start. Mr. Gibson's efforts on her behalf were, however, tireless. The trial began on October 7. Mr. Gibson records that Miss Cavell "made a signed statement admitting the truth of the [above] charges" and had further admitted that some of those whom she had assisted "had written her from England thanking her for her assistance." This admission, whatever we may think of its courage, made her case serious. Article 58 of the German military code, under which she was tried, says that any person guilty of "conducting soldiers to the enemy" will be "sentenced to death for treason."

### WHAT THE DEFENCE WOULD HAVE BEEN.

Mr. Gibson adds that this provision was manifestly strained against Miss Cavell, since to help soldiers to enter a *neutral* country in the hope that they might regain their regiments was not foreseen when the code was drafted. The law was framed to cover those who helped stragglers to get back to their own lines. The neutral country to which Miss Cavell helped soldiers to escape was, he points out, bound to apprehend them. If it did not, that could be no affair of hers. In court, with great courage, Miss Cavell admitted the charge, and gave as her reason a fear that the men, unless helped to escape, would have been shot. When all pleas for clemency had proved in vain, when every attempt to delay proceedings had failed, when the Germans turned a deaf ear to the reminder of the care which Miss Cavell had bestowed on German wounded, when attempt after attempt to appeal to the Kaiser had been vetoed, it was discovered that "the sentence on Miss Cavell was not pronounced in open court," but actually in her cell, and, as we all know, "she was taken out and shot before daybreak."

### "PATRIOTISM IS NOT ENOUGH."

That she had committed an offence is true, that this offence was enhanced by the neutral profession to which she belonged is also true; but that her efforts were stopped, and that justice would have been satisfied, by her imprisonment, that the procedure of her captors from the moment of her arrest until her death was an abomination, and that the spirit in which she met her death was noble are true also. "Patriotism is not enough," is her last recorded word: "I must have no hatred or bitterness towards anyone." The first four words are her real epitaph. With finest discrimination, they give to the world her own verdict, when through trouble she had plumbed herself. They alone are fitting to be written below the statue on which Sir George Frampton is at work. The remaining sentence, "I must have no hatred or bitterness" is a lesson which her spirit presumably wishes us to learn. For the facts and their telling *Land and Water* deserves the public's thanks. Mr. Gibson's own efforts for Miss Cavell have won the gratitude of Englishmen.

### A SUPERMAN AT THE METROPOLITAN HOSPITAL.

A hospital's "superman," who is on duty twenty-four hours at a stretch, was described to the Hackney Tribunal the other day when Mr. Buchanan, the secretary and house governor of the Metropolitan Hospital, appealed for the exemption of William Randall, the institution's porter-attendant, classified C1. Mr. Buchanan stated that in normal times they would have had seven men to do the work Randall and his cousin, the mortuary keeper, now did. He attended 300 wounded soldiers, saw to the surgery instruments—which was skilled work—and part of his duty in the theatre was to assist in getting patients under the anæsthetic, and in certain cases this required some strength. He was the only man available for obstreperous cases. Randall was always on duty; in fact he was a twenty-four-hour man. The military representative objected that Mr. Buchanan was making Randall out to be a superman, and Mr. Buchanan replied, "I am. He is one—a hospital superman, at any rate." The Tribunal gave the superman three months' unconditional exemption.

### THIS WEEK'S DRUG MARKET.

While actual changes in value of any particular importance are few in number, the tendency of prices is still in an upward direction, although there are several exceptional cases of drugs which are tending lower in value. Thus benzoic acid and sodium benzoate are offered at materially lower prices; the prices of salicylic acid and sodium salicylate are by no means so firmly maintained; phenacetin is offered at rather lower prices, and the same may be said of resorcin and phenolphthalein. Cinchona bark is dearer; quinine is also inclined upwards in price, and the demand is rather more active. Star aniseed oil and cassia oil are again dearer. Honey is in good request, and the price tends upwards. Saccharin is still very scarce, and the extreme values are well maintained. Olive oil is as scarce as ever, and liquorice juice is in small supply and exceedingly dear. Good prices have been paid for gum benzoin.

